# Closure of the Vagina

**Published:** 1828-12

**Authors:** 


### HOSPITAL REPORTS,

*(Principally condensed from various Periodical Publications.)*

### CLOSURE OF THE VAGINA.

### *Closure of the Vagina after difficult Labour ; Operation.*

Ann Smith, twenty-six years of age, was admitted into St. George's Hospital on the 3d of September last, with stricture of the vagina, succeeding protracted parturition. On examination it was found that an aperture existed, barely admitting the smallest bougie, and apparently owing to a very large cicatrix, the contraction of which had narrowed the opening. From this small aperture there issued much discharge, of brownish colour and offensive odour. The urethra had become so widely dilated as readily to allow the little finger to pass into the bladder. She stated that, two years before her admission, being some months gone with child, she was suddenly taken in labour, which lasted two days and a night; after which it was discovered that a portion of the vaginal membrane protruded through the os externum, and in the course of a few days it sloughed away. Bearing-down pains were felt for some time, and the urine occasionally came away in a very large stream.

The bowels having previously been emptied by aperients and an enema, Mr. Keate, on the 11th, divided the stricture by means of the bistoure cachée, the incisions being carried upwards towards the arch of the pubes. The forefinger was then introduced into the opening, and the os uteri found unaffected. The discharge on breaking down the barrier of membrane was horribly fœtid.

Three hours after the operation the patient experienced a rigor. The vagina was then syringed out; two or three coagula removed; a piece of oiled lint introduced; and five grains of calomel ordered immediately, followed soon afterwards by a dose of castor oil. Salines, with the sulphate of magnesia, every six hours.

From this time no febrile affection, or other constitutional disturbance, was experienced, an occasional purgative only being needed. On the 17th, the discharge was become purulent and healthy; and on the 22d, a bougie, three-quarters of an inch in diameter, was introduced into the vagina, after which she commenced the use of the dilator.

A little pyrexia and headache took place on the 2d of October, but were readily removed by salines, with antimony and a senna draught. On the 5th, when we saw her, she was well enough in health, and could bear the introduction of a good-sized uterus bougie into the vagina.

